# A Sight to Wheat Bran: High Value-Added Products

**DOI:** 10.3390/biom9120887

**Published:** 2019-12-17

**Authors:** Agne Katileviciute, Gediminas Plakys, Aida Budreviciute, Kamil Onder, Samar Damiati, Rimantas Kodzius

**Affiliations:** 1 Panevezys Faculty of Technology and Busines, Kaunas Technology University (KTU), 37164 Panevezys, Lithuania; akatileviciute@gmail.com (A.K.); gediminas.plakys@gmail.com (G.P.); aida.budreviciute@gmail.com (A.B.); 2 Procomcure Biotech, GmbH, 5304 Thalgau, Austria; oender@procomcure.com; 3 Department of Biochemistry, Faculty of Science, King Abdulaziz University (KAU), Jeddah 21589, Saudi Arabia; sdamiati@kau.edu.sa; 4 Faculty of Medicine, Ludwig Maximilian University of Munich (LMU), 80539 Munich, Germany; 5 Mathematics and Natural Sciences Department, The American University of Iraq, Sulaimani (AUIS), Sulaymaniyah 46001, Iraq

**Keywords:** wheat, wheat bran, by-product, renewable, agriculture, biotechnology, enzymes, biochemicals

## Abstract

Recently more consideration has been given to the use of renewable materials and agricultural residues. Wheat production is increasing yearly and correspondingly, the volume of by-products from the wheat process is increasing, as well. It is important to find the use of the residuals for higher value-added products, and not just for the food industry or animal feed purposes as it is happening now. Agricultural residue of the roller milled wheat grain is a wheat bran description. The low-cost of wheat bran and its composition assortment provides a good source of substrate for various enzymes and organic acids production and other biotechnological applications. The main purpose of this review article is to look into recent trends, developments, and applications of wheat bran.

## 1. Introduction

By following the Food and Agriculture Organization of the United Nations (FAO), cereals, including wheat, rice, barley, maize, rye, oats, and millet, make up the major part of crop production. They extend to be the paramount nutrition source for human intake [1]. According to FAO statistical data wheat production reached 772 million tons in 2017 and it is expected to increase by 2.5 percent annually [2].

**Figure 1:**
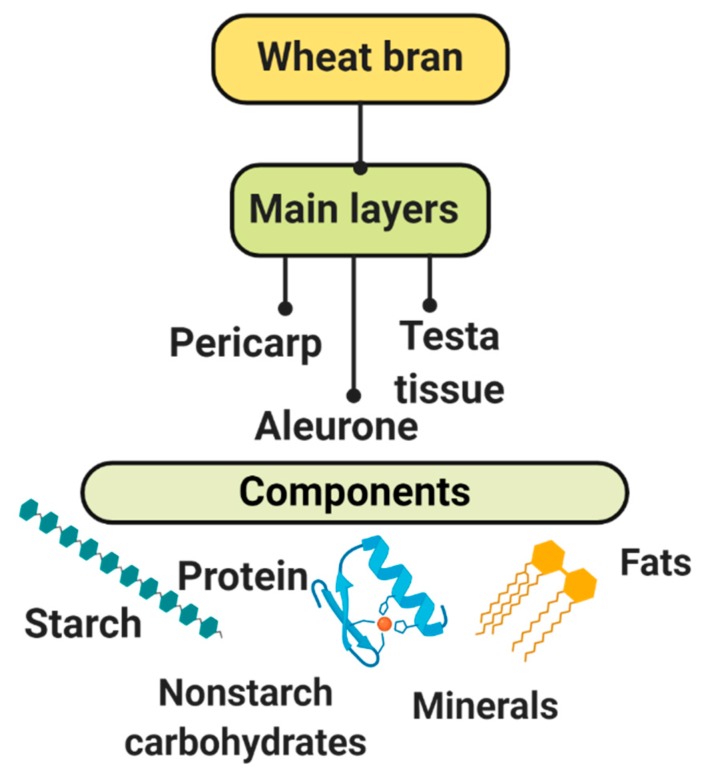
Wheat bran layers and components distribution. These constitute 55–60% of nonstarch carbohydrates, 14–25% of starch, 13–18% of protein, 3–8% of minerals, and 3–4% of fat calculated on a dry matter [Created with BioRender.com].

Wheat grain is composed of different tissues: the germ, endosperm, aleurone layer, and pericarp [3]. Wheat grain is rich in bioactive compounds, micronutrients, and phytochemicals. A higher concentration of these components is located in bran fractions. Wheat bran (WB) is a residue of the rolled milled wheat grain [4]. The main layers of WB are pericarp, aleurone and testa tissue [5]. The components distribution in WB are as follows: 55–60% are nonstarch carbohydrates, 14–25% is starch, 13–18% of protein, 3–8% of minerals and 3–4% of fat, measured on a dry matter (Figure 1) [6].

The nonstarch carbohydrate fraction is composed of 52–70% of arabinoxylan [6]. A quarter ton of WB comes from one million tons of wheat. Around 150 million tons of WB are made during year worldwide, and its main application is as a low-worth ingredient in animal feed [7]. The huge amounts of the WB by-products containing all the components of interest can be used to isolate the components or convert these in various ways, such as separation by fractionation or extraction, fermentation and many other ways

An interest in renewable energy sources, such as wind, solar, geothermal energy, hydro, biofuel, and agricultural by-products applications has been increasing in recent years. Agricultural by-products like WB appear as a promising feedstock for obtaining high added-value products (Table 1; it is also a low-cost residue that makes it cost-effective [8].

Microorganisms cultivated on agro-industrial residues serving as a food source can supply several other useful products including enzymes, organic acids, chemical additives, pigments, food additives, antibiotics, biofuels, solvents, and bioplastics [36].

Bacteria and fungi grown on agro-industrial by-products are essential/relevant sources of enzymes used in food-biotechnology, pharmaceutical, animal feed, paper industries, and textiles. According to FAO, the increasing need for economical production methods, new functionalities, increasing safety requirements, and reduction for environmental impact leads the trend toward the alteration of traditionally chemical procedures with enzyme-based reactions [36]. Various biochemicals are being used in pharmaceutical, cosmetic, food, leather, and textile industries. Nowadays natural and ecological production is gaining popularity in chemical synthesis manufacturing [37], agro-industrial biowastes being one of them. Agro-industrial biowastes can be a great source for bioremediation, i.e., heavy metals ion fixation or removal, alternative production of oligosaccharides, single-cell oils, bioplastics, biofuel, biosurfactant production, as well as for the cell immobilization. The industrial by-product processing could be applicable to obtain various products from WB.

## 2. Enzyme Production by WB Utilization

Enzyme structure usually consists of proteins that are folded into various complicated special 3D shapes. The primary function of enzymes is to act as a catalyst to accelerate various reactions. Enzymes are grouped into six classes corresponding to the type of reaction catalyzed: transferases, oxidoreductases, lyases, hydrolases, ligases, and isomerases. They are widely used for commercial and industrial purposes. The key areas of enzyme application are pharmaceutical, food, and cosmetic industries [38]. WB is a cheap agro-industrial by-product and a good source of substrate for cultivating various microorganisms. Microorganisms use WB mainly as a source of carbon (C) and nitrogen (N). The enzymes reviewed are presented in Table 2.

There are two main methods to deliver enzymes—either by enzyme production by solid-state fermentation (SSF) or by submerged fermentation (SmF). Classification into SSF and SmF is principally based on the sample of substrate used through fermentation (solid or liquid state).

### 2.1. Enzymes Production by SSF

Enzymes produced by SSF method utilize the increase of microorganisms on water-insoluble substrates without free water [59,60]. All solid substrates have a principal macromolecular composition consisted of starch lignocellulose, cellulose, pectin, and other polysaccharides [61]. Substrates for SSF are heterogeneous products or by-products from the agriculture industry. Agitation is not applied in majority aerobic SSF procedures, especially in static reactors, such as tray fermenters. Agitation is generally a relevant part of continuously or periodically agitated SSF bioreactors [62]. As previously described, aeration accomplishes four principal functions in SSF, such as sustaining aerobic conditions, desorbing carbon dioxide, controlling the substrate temperature and the humidity level [61]. The technique is efficient and gives a higher production yield than submerged cultures, also inoculum ratio is always larger and agitation may not be necessarily used. The essence of this fermentation technique is that the substrates are used very gradually and steadily; therefore, the same substrate can be used for long fermentation periods. Consequently, in this technique the release of nutrients is controlled and maintained. SSF is most appropriate for fermentation methods with fungi and microorganisms involved, which require a lower humidity content. Despite that, organisms that need a large amount of water for their activity, such as bacteria, cannot be used with this fermentation process [63].

The possibility to utilize the agricultural waste for xylanolytic enzyme production was explored by Ferreira et al. [39]. Sugar cane, corn cob, bagasse, and WB were tested as substrates for xylanolytic enzyme production in SSF by *Aspergillus tamarii*. High proteolytic activity was observed in WB cultures, while weak proteolytic activity was discovered in sugar cane bagasse and corn cob cultures. The largest l-xylosidase and xylanase activities were obtained 1.5 times faster with WB cultures than with other cultures used. Furthermore, the optimal moisture content of the media was 86% with WB, while for corn cob and sugar cane bagasse were 80% and 75% respectively. Nagar and others found elevated production of cellulase-poor alkali stable xylanase by *Bacillus pumilus* SV-85S in the presence of WB under SSF [40]. The enzyme was entirely stable over a wide pH (5–11) interval and maintained 52% of its efficacy at a temperature of 70 °C for 30 min. Approximate evaluation of price collation showed that the price of the enzyme generation using WB was reduced to 50%. It is well known that the pectinase, xylanase, α-amylase, and compounds belonging to saccharification content (total soluble carbohydrates and reducing sugars) are produced by *Bacillus megatherium*. The production was tested in diverse SSF samples, such as WB, palm leaves, grasses, and date seeds [41]. The peak production of xylanase, pectinase, and α-amylase was obtained (150, 350, and 100 units/g solid, respectively) with WB substrate, in comparison with other agricultural residual. One of the main advantages of using WB is that WB itself contains enough nutrients and no additions of carbon and nitrogen sources are needed.

WB was selected as an effective substrate for milk-clotting enzyme production by three strains of *Bacillus subtilis* [57]. The optimized medium possessed WB (30 g/L), glucose (16.2 g/L), NaCl (5 g/L), MgSO_4_·7H_2_O (5 g/L), KH_2_PO_4_ (2 g/L), and CaCO_3_ (3 g/L). WB was used as the single nitrogen source in the media and the slow release of nitrogen from WB is thought to support bacterial growth [64]. This agro-industrial by-product includes glucose that in most cases is vital to the microorganisms for growth and metabolism support. For these reasons, WB discovered to be the most appropriate substrate for polygalacturonase (PG) production. No addition of any nutrient or stimulating supplement was needed, either. PG was produced by *Aspergillus sojae* mutant strain [58]. l-glutaminase was produced by *Vibrio costicola* using WB with particle sizes from 1.4 to 2.0 mm [54]. The preferable substrate for l-glutaminase manufacturing in SSF was WB [52]. Kashyap et al. investigated the glutaminase production from *Zygosaccharomyces rouxii* NRRL-Y 2547 by SSF using WB and oil cake from sesame [53,55]. El-Sayed reasearched the same enzyme production by *Trichoderma koningii* using WB as solid support. He could demonstrate that after optimization, l-glutaminase productivity by the solid cultures of *T. koningii* grew up by 2.2 fold concerning to the submerged culture [56].

Different combinations of WB and soybean were tested for the growth of *Xylaria nigripes* (XN) by SSF [65]. XN mainly used for insomnia and trauma treatment, as well as a diuretic or nerve tonic. When WB was used as a single substrate, the ethanol extract of XN-fermented matter achieved the highest antioxidant activities. Combining the equal amounts of WB and soybean for SSF helped to increase the protective impact against H_2_O_2_-stimulated lesion in neuronal cells (PC12 cells) [65]. The conclusion was that both substrate remnants of fermentation may have an impact on the biological activities of XN-fermented substances [65].

*Pseudomonas aeruginosa* was tested for alkaline protease production by using WB via SSF [42]. The main fraction of the WB aleurone layer is composed of 50% of phytic acid [43]. Its degradation makes beneficial supplies for easily digestible fiber foods. Therefore, WB can be used as phytate sources in the fermentation to expand phytase activity [44,45]. Salmon et al. reported the phytase production by *Schizophyllum commune* in SSF with the substrate as WB, and showed 96 U/g at 66 h in the sequel optimization studies that were performed [45].

### 2.2. Enzyme Production by SmF

In SmF, the substrate used for fermentation is always in the liquid state containing the nutrients needed for the culture growth. This fermentation method is best suitable for microorganisms like bacteria that need water for growth [66]. One of the major benefits of this fermentation type is the straight forward purification of the product. It is widely used for the extraction of secondary metabolites that are secreted into the growth medium [67]. Five different agricultural waste sources were selected as a substrate and WB was found to be the most appropriate for co-production of alkaline protease and xylanase from *Bacillus licheniformis* NRRL 14209 using Box–Behnken Design under SmF [68]. Another research demonstrated that the highest enzyme efficiency of xylanase (4.31 U/mL) and alkaline protease (3.66 U/mL) was gained at 24 h of the incubation duration, primary media pH 8.5 with 0.5% *w*/*v* WB and 4% (*v*/*v*) inoculum concentration at the temperature of 30 °C [48]. Ferulic acid esterase (FAE) production was carried out using *Streptomyces S10* culture and destarched wheat bran (DWB). After optimizing the conditions, the enzyme yield reached 2.0 mU/mL in MBS medium, which contained 1.5% of DWB under the agitated submerged culture [69]. Carboxymethyl cellulase expression in *Aspergillus flavus* was optimized with culture conditions at an optimum pH of 6.0, temperature of 30 °C, inoculum size of 4% in Czapek Dox using WB as a substrate by SmF. Other substrate options were: cotton seeds, pomegranate, rice bran, and rice straw. Enhanced production occurred with the addition of 4% of WB and 1% of peptone as nutritional factors [49]. WB was used as low-cost lignocellulosic support for fungus growing and laccase manufacturing by *Cerrena unicolor* C139 in SmF. It was demonstrated that the laccase generation begin after 2 days of cultivation, achieved the highest activity of 416.4 U/mL after 12 days of fermentation [70].

Nampoothiri et al. explored thermostable phytase generation by *Thermoascus auranticus* in SmF and 3.75% (*w*/*v*) WB particles as carbon origin complemented with sucrose, glucose, peptone, starch, and minerals [46]. A 4.5-fold enzyme activity increment was observed. Sarsan and Reddy investigated the equal substrate, for phytase production by *Bacillus* sp. C43 in SmF appended with glucose and sucrose, which achieved 0.52 and 0.59 U/mL of phytase activity, accordingly [47]. Laccase production by *Trametes modesta* was successfully optimized using a one-factor-at-a-time method. WB, yeast extract and incubation temperature appeared to be the main factors influencing laccase production by *T. modesta* [50]. The same study showed that the laccases from *T. modesta* showed their maximum activity at pH 4 and at 50 °C and were steady at pH range 5–6 and at 40 °C [50]. Efficiently produced laccase was isolated from fungus *Trametes* sp. LS-10C and the laccase producing medium was optimized by the response surface methodology in shake flask fermentation [51]. WB diffusion juice was selected as one of the optimized medium ingredients for the scale-up fermentation [51].

Overall, good yields could be obtained with the general conditions of pH (4–11), the temperature of 30–70 °C, the cultivation medium should contain glucose, sodium and magnesium salts, fermentation time can vary from 2 till 12 days, substrates combination of WB and soybean can also provide good results.

## 3. WB as a Source for Organic Acids Production

It is widely known that microbial strain selection is a highly relevant factor in the production of organic acids [2]. Microorganisms used must possess steady characteristics, grow quickly and strenuously, be non-pathogenic and generate high yields of the desired product according to FAO [2]. Wheat is rich in phenolic acids: ferulic acid, syringic acid, *p*-coumaric acid, caffeic acid, and vanillic acid [71]. It was already demonstrated that the phenolic compounds are differently spread in wheat grain tissues [72]. Furthermore, together with phenolic acids, polyphenols such as lignans can be found on the living-cell aleurone layer, but concrete phenolics are located in bran fractions [73]. Bound phenolics are the major group in wheat grains and have been found to possess the highest antioxidant activity. The same study identifies ferulic acid as the most plentiful compound in the bound form [73]. Another widely used acid is lactic acid. This acid is mainly produced by bacterial fermentation of starch, involving biomass from renewable supplies as WB. Recently, there has been an increased interest in the application of renewable material for the production of various chemicals. Two organic acids, the ferulic and lactic acids, have a significant part in various industries and have been produced from WB.

### 3.1. Ferulic Acid (FA)

**Figure 2:**
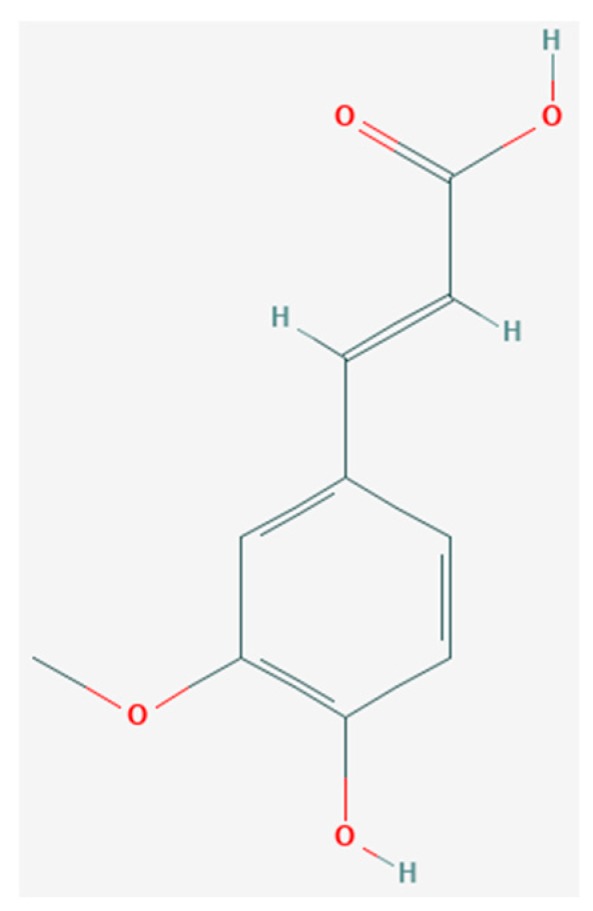
Ferulic acid chemical structure [78].

Ferulic acid (Figure 2) is mostly used in pharmaceutical, cosmetic, and food industries [74]. Moreover, this acid covers plenty of potential medical implementations, as a scavenger of free radicals or as a protective agent against UV radiation-induced skin harm [75,76]. It was demonstrated that the FA is mainly bound in the cereals bran in the form of ester linkage [77]. Based on that various methods have been studied for FA release from their ester-linked compounds.

The mixture of flax shives, corn, and wheat bran was used for FA extraction and purification by non-pressurized alkaline hydrolysis with 0.5 M NaOH and pressurized solvents (0.5 M NaOH, water, ethanol, and ammonia) [79]. There were no differences in the content of products extracted with non-pressurized and pressurized 0.5 M NaOH solution yielding mostly FA, *p*-coumaric acid, and small amounts of vanillin.

Xie et al. studied edible mushrooms that are capable to release FA from WB. Here *Hericium erinaceus* produced the maximum FA yield at 4 days of culture, and reached 95.51 mg/L in WB broth [76]. FA was released from DWB under the action of AnXyn11A and AnFaeA [80]. Gopalan and Nampoothiri also used DWB for the purification and to find it as a good adsorbent for the FA [81]. Dupoiron et al. studied downstream purification of the WB enzymatic hydrolysate. Hydrolysis was carried out with hemicellulasic cocktails received from *Thermobacillus xylanilyticus*. The purification process was carried out with a weak anion-exchange resin (Amberlyst A21-Dow) in a free-base form. 52% of FA was released from WB [82].

### 3.2. Lactic Acid (LA)

**Figure 3:**
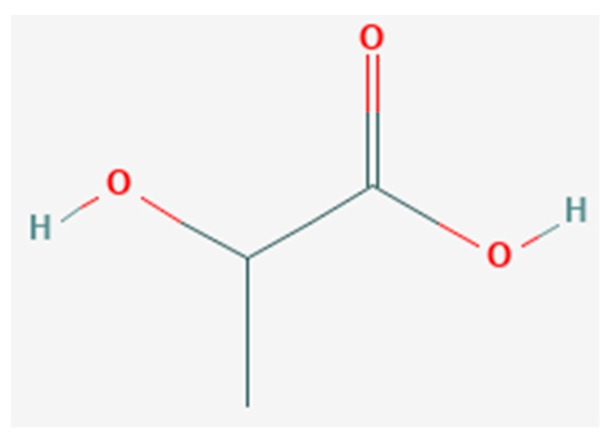
Lactic acid chemical structure [86].

LA (Figure 3) is one of the first noted fermentation products from microbial metabolism with the structure of two enantiomers: synergistic L (+) and D (−) [83]. LA and its derivatives are broadly used in food, pharmaceutical, leather, and textile industries [84]. Acid is produced by chemical synthesis and microbial fermentation [85].

LA production from WB has been announced by Naveenaet al [87]. The authors tested different bran for LA production by *Lactobacillus amylophilus* GV6. Different bran (pigeon pea, green gram, black gram, corn, and WB) for LA production by *Lactobacillus amylophilus* GV6 were studied. WB was found as the best solid support and substrate from all the other ones. The same *Lactobacillus* strain was used for Plackett–Burman design [88] and screening of 15 parameters for the production of L (+) LA from WB substrate and solid support. “The nitrogen sources, peptone, yeast extract, and tri-ammonium citrate, along with NaH_2_PO_4_ H_2_O and Tween 80, were found to increase productivity” [83]. Yun et al. announced the production of LA from rice and WB hydrolyzate, without additional nutrients by the batch culture of the isolated LA bacterium *Lactobacillus* sp. *RKY2* [89]. Experiments confirmed that fermentable carbohydrates and nutritional factors from rice and WB might be an effective nutrient for LA fermentation. 

### 3.3. Other Acids Production Utilizing WB as a Source

There are some other organic acids that can be derived from WB. The industry did not realize until now a wide range of organic acids production from WB, and the publications on this subject is scarce. Besides FA and LA, the itaconic acid (IA) and fumaric acid are the main organic acids produced from WB as a base.

The main applications of itaconic acid (2-Methylidenebutanedioic acid) are in medicine, the chemical industry, agriculture, and the industrial production of acrylic acid, resin plastics, latex, acrylate, slush powder, and anti-scaling agents [90,91,92,93]. Production of IA was investigated by the biotransformation from WB hydrolysate. The IA yield was further increased by utilizing the *A. terreus* mutant strain CICC40205 [94].

Another important acid for medicine, polymerization and esterification reactions is fumaric acid [95]. Related to that, a study was performed where WB was utilized as feedstock to synthesize fumaric acid by *Rhizopus oryzae* [96]. WB pretreatment with sulfuric acid hydrolysis at 100 °C for 30 min was the optimal choice for fumaric acid production. Vanillin is one of the most widely used flavours in the food industry. Vanillin was obtained from the bioconversion of FA-derived from enzymatic hydrolysis of wheat bran [97].

## 4. Biotechnological Applications of WB to Environmental Treatment

The main field of WB conversion still remains food sector. This wheat milling waste can be used in environmental remediation, as well. WB as an adsorbant is a great source for biodegradation, bioremediation, or bioabsorption process.

### 4.1. Biodegradation Process

The biotic degradation or biotic decomposition chemical of contaminants by bacteria or other biological means [98]. Various substrates are used as a natural filter for decontamination of an industrial effluent containing heavy metals, inorganic chemical and other hazardous waste compounds [5]. The first step in biodegradation is to adsorb material and then to degrade it by specific microorganisms. Since WB made up from lignin, cellulose, and fatty acid units whose functional groups (hydroxylic, carboxylic, and phenolic) are perfect for ion fixation [99]. Organic material can be degraded aerobically or anaerobically. Important factors for bioremediation are microbial populations that are metabolically efficient and sustainable, appropriate environmental growth conditions, temperature, the presence of water, and favorable acidity or alkalinity [98,100].

The triphenylmethane dye malachite green (MG), is used as a fungicide and antiseptic in fish cultures, direct dye for silk, wool, jute, and leather. It is toxic to bacteria and mammalian cells [101,102]. MG was adsorbed onto WB with a particle size of 8–20 mesh by using a batch technique. MG degradation process was carried out by *Fomes sclerodermeus* at pH 5, because these fungi are able to convert lignin to inorganic material [101].

Free gossypol (FG) is a yellow coloring pigment present in cotton [103]. It causes the decrease of animal growth and feeds conversion and depression of fertility in bulls and reduction of viability of gametes in cattle [104,105]. Wen and Sun evaluated *Candida tropicalis* ZAU-1 ability in biodegrading free gossypol by analyzing the time course of SSF. The solid medium contained 20% of WB. It was the second-largest component in media after cotton seed (60%) [103]. As expected, *Candida tropicalis* biodegraded the FG up to 3.55% and 21% of dry matter of substrate (mixture of cottoseen, WB, rice bran and rice wine spent grain) were lost after 64 h of SSF.

We could expect further use of WB for the biodegradation of various other, even potentially toxic biomaterials.

### 4.2. Wheat Bran as Biosorbent

While in the biodegradation process the WB acts as biosorbent, and the final absorbed material is degradated, the WB can act as biosorbent for various inorganic and organic toxic substances, which not necessary can be degradated (such as heavy metal ions). WB is a great source for biosorption process because it contains large amount of cellulose and ligin which works as the adsorbent material [7,101]. Toxic elements ions such as Cr, Fe, Se, V, Cu, Co, Ni, Cd, Hg, As, Pb, and Zn are of special attention regarding their toxicity, bio-accumulation tendency, and persistence in nature [106]. As stated in the study, these elements are not biodegradable and have a tendency to accumulate in live organisms, inducing different diseases and disorders [107]. 

The potential use of rice and wheat bran was for sequestering cadmium and significant removal efficiency was reported [23,108,109]. Bulut et al. investigated the adsorption of Pb(II) ions from aqueous solutions on WB [110]. They found that adsorption of Pb (II) onto WB is an endothermic and spontaneous process, also the Pb (II) amount decreased with increasing dose of adsorbent, pH, and temperature, and the best suitable particle size of milled WB for adsorption process would be 500 μm.

WB has been found to be an economically usable and efficient biosorbent for the removal of Cr (VI) [22]. Here, the highest removal of Cr (VI) was achieved to be 310.58 mg/gat pH 2.0, initial Cr (VI) concentration of 200 mg/L and temperature of 40 °C [22]. Kaya et al. used WB and modified WB (M-WB) for Cr (VI) removal [24]. The authors realized the chemical modification by using citric and tartaric acids. WB and M-WB have removed 4.53 mg 5.28 mg of Cr (VI)/g from the solution accordingly [24].

Microbes bound to a WB (85%)/red wood powder (10%)/diatomaceous (5%) earth carrier were used as inoculants for a biotrickling filter. The filter is a combination of a biofilter and a bioscrubber, where bacteria responsible for decomposition are immobilized on a carrier or filter material [111] for contaminated gas treatment with a combination of benzene, toluene, and o-xylene [112]. WB was used for a natural biosorbent preparation with multiple quaternary ammonium salts This natural biosorbent was used for removal of dye (AR-18) from aqueous solution [113]. Polylactic acid (PLA) and several plasticized PLA based systems were biodegradated by *Trichoderma viride* fungus, in a liquid medium and controlled laboratory conditions [114]. WB was a carbon source in a liquid medium.

## 5. Further Applications of WB in Biotechnology

Besides biodegradation, bioremediation, or bioabsorption processes, there are many other well-suited applications in the industries where WB is or can be utilized. WB as a cell immobilization carrier is an excellent source for the feruloyl oligosaccharides, wheat bran oil extraction, single-cell oils (SCO), and polyhydroxybutyrate (PHB) production.

Yuan et al. identified xylanases from *Bacillus subtilis* able to hydrolyze WB for the production of feruloyl oligosaccharides. The optimum WB concentration was 120 g/L, with 42 °C, pH 5.2. After 35 h inoculation, enzyme concentration was 4.8 g/L and substrate concentration, 120 g/L. The resulting concentration of various oligosaccharides was measured by specrophotometer and determined to be 1.5 mM for the 120 g/L substrate (wheat bran insoluble dietary fibre) [115]. The authors state that the “isolation of these feruloyl oligosaccharides enabled a better understanding of the plant cell wall structures” [115]. Another study demonstrates the use of mesoporous silica catalysts, which was found as a good option for the hydrolysis of real arabinoxylans derived from WB [116].

Microbial oils, called SCO, are used for commercial applications as nutraceuticals, pharmaceuticals, and feed ingredients for aquaculture [117]. *Microsphaeropsis* sp. was used for the production of SCO in SSF from a substrate consisting of steam-exploded wheat straw and WB [118].

WB was used as a cell immobilization carrier for probiotic yogurt production by *L. casei* strain in combination with *L. bulgaricus* strain. The WB delignification by alkali treatment enhanced viability of both strains and helped to maintain high viable cell numbers through storage at 4 °C [119]. Delignified WB was used for the immobilization of *L. paracasei* K5 cells and used for functional Cornelian cherry beverage production with potential synbiotic properties [120]. Xie et al. reported about in situ fortifications of vitamin B12 in wheat flour and WB by fermentation with *Propionibacterium freudenreichii* [121].

It is well known that the PHB is an organic polymer with commercial potential as a biodegradable thermoplastic and a biomaterial [122]. Alkaline pretreated WB was enzymatically hydrolyzed using cellulase of *Trichoderma reesei* and glucosidase of *Aspergillus niger* and then used for the production of PHB [123]. Zhang et al. demonstrated that WB can be an efficient and sustainable raw material to receive low-cost carbon products with a high surface area and an indication of its potential utilization [124].

Surfactants are materials used to decrease surface and interfacial tension in various industrial processes. According to FAO, agro-industrial by-products with a high carbohydrate or lipid content can be used as substrates for the production of biosurfactant [2].

With the increasing amount of WB production, we may expect other uses of WB in biotechnology. The WB content may change depending on the wheat strain used, opening new ways for WB utilization.

## 6. Future Perspectives for WB Application

According to FAO, within the cereal group, it is expected worldwide that relative significance of rice is going to decrease slightly, while wheat consumption will continue to grow in per capita terms [125]. Right now, the utilization of WB is still focused on the sectors of food and feed supplement. Many researchers demonstrated that this renewable resource could be used in various ways. It is known that WB consists of quite a large percentage of proteins (8–12%) [8]. Because of that, WB is a great source for protein extraction. Extracted and purified proteins could be used for plant-based nutrition which is highly popular these days. Extracted proteins could be used for amino acid production and potentially included in athletes’ diet, as an ingredient in sports drinks and supplements.

There is also an increased interest in safer drug forms. The plant polysaccharides are of special interest as WB consist of more than 50% of nonstarch carbohydrates. Prisenžnakova et al. revealed that arabinoxylan type WBH1 had great pharmacotherapeutic potential in the therapy of cough but still there is no information about WBH1 clinical use in cough therapy [126]. WB is a rich source of fatty acids, tocopherols, and phenolic compounds [3]. Therefore, it could serve as a natural source for vitamin production. Various agro-industrial byproducts can also be used as substrates for medical-grade or edible mushroom production [127]. We conclude WB has a good impact on health, therefore we can expect further development of this field and increased dedicated funding to the research. It might be one of the keys to preventing or even to stop serious diseases.

Nowadays the cosmetic industry is trying to create as many as possible natural and environmentally friendly products, containing ingredients extracted from natural sources. Grain cultures are already widely used in the cosmetic industry, but WB is unique, with the presence of specific oils, fats, and antioxidant activity. The area of interest could be a skin conditioning agent, humectant, exfoliate, or anti-aging agent.

There are lots of reports on biofuel production from WB, but in practice, it mostly remains on a laboratory scale. There is a potential to commercialize biofuel originating from WB. A scale-up of starch-based biofuel could substitute the use of fossil fuel, enabling a greener and sustainable future.

With the constant worldwide human population growth, food production increases. The wheat production increases as well, over recent years, resulting in WB higher amounts. While this is still used for the animal feed, there is a clear trend towards high value-added products. We may expect that some components produced from the WB will diminish in the future (such as food, metabolite production, heavy metals removal), while the higher value-added products will increase (such as health-related drugs, enzymes). The WB role in biofuel production may increase shortly, but we consider it as non-sustainable, as both WB and biofuel are based on the carbon source. Therefore, in the future its role in biofuel may diminish. In the end, to diminish the waste, all the WB should be efficiently utilized, retrieving the highest value products, then using the remaining fractions as the lower value input. Ideally, the waste conversion should be located near or in the plants producing that waste, ultimately leading to zero-waste production.

## Figures and Tables

**Table 1:** The role of wheat bran in various fields.

Field	Application/Product	Role as/in	References
Enzymes	Enzyme production by solid-state fermentation (SSF) and submerged fermentation (SmF)	Substrate for enzymes production	[5]
	As an inducer for enzymes	Complex substrate	[9,10,11]
	Production of protease, amylase, and glucoamylase	Nitrogen source	[5]
Metabolites	Bacitracin Cyclosporine-A Gibberellic acid	Cheap raw material	[12,13,14]
Biofuel	Bioethanol Biobutanol Biohydrogen	Lignocellulosic material	[15,16,17,18,19,20,21]
Heavy metals removal	Removal of, Hg (II), Cd (II) Pb (II), Cu (II), Cr (VI), Ni (II)	Biosorbent material, lignocellulosic substrate	[22,23,24]
Health	Minimizes the risk factor for various illness: Diabetes, colon cancer, hypertension, coronary heart disease	Fiber source, strong antioxidant activity, bioactive agents that inhibit colon carcinogenesis	[25,26,27,28,29,30,31]
Food	Enrich the nutritional and physical properties of bread and baked products;	Nutritional and physical properties	[32,33,34,35]
Feed additive	The stock material for animal feed preparations	High starch content, indispensable amino acids, high content of non-starch polysaccharides	

**Table 2:** Enzymes production by wheat bran (WB) utilization in solid-state fermentation (SSF) and submerged fermentation (SmF).

Fermentation Method	
SSF	SmF
Produced enzymes	Produced enzymes
Xylanase [39,40,41]	
Alkaline protease [42,43]	
Phytase [44,45,46,47]	
l-xylosidase [39]	Ferulic acid esterase [48]
α-amylase [41]	Carboxymethyl cellulase [49]
Pectinase [41]	Laccase [50,51]
l-glutaminase [52,53,54,55,56]	
Milk-clotting enzyme [57]	
Polygalacturonase (PG) [58]	
